# Development of the Tunneled Agar Plate Assay (TAPA) for Microbial Co-culture Screening of Antibacterial Activity against Pathogenic Bacteria

**DOI:** 10.4014/jmb.2608.08043

**Published:** 2026-10-14

**Authors:** Gyeongsik Shin, Hyuntae Kim, Yujeong Kim, Junho Lee, Juyoung Kang, Eunyeong Yun, Taeseop Song, Seunghyeon Ha, Jinhyeok Hur, Haein Lee, Sol Kang, Chaeyeon Kim, Siwon Choi, Jeongmin Park, Garam Kim, Jeongho Lee

**Affiliations:** Department of Biosciences and Bioinformatics, Myongji University, Yongin 17058, Republic of Korea

**Keywords:** Co-cultivation, Antibacterial screening, Microbial interaction, Natural products, Tunneled agar plate assay, 3D printing

## Abstract

Microbial co-cultivation is a powerful strategy for unlocking cryptic antibacterial metabolites. However, many existing platforms remain technically demanding and do not integrate co-cultivation with pathogen growth-inhibition screening in a single high-throughput assay. Here, we present the tunneled agar plate assay (TAPA), a 3D-printed 96-well device. TAPA spatially separates co-cultured microorganisms into distinct wells while permitting metabolite diffusion through a shared agar matrix, enabling simultaneous co-culture and antibacterial screening against pathogenic bacteria. Proof-of-concept experiments with ampicillin, aztreonam, and imipenem, together with environmental isolates of known inhibitory activity, confirmed that TAPA reliably detects diffusion-mediated growth inhibition. Visual assessment closely matched quantitative MTT assay results. TAPA was then applied to an in-house library of environmental bacteria, testing all 6,889 pairwise combinations among 83 isolates against *Staphylococcus aureus* and identifying 106 screening hits that exhibited inhibitory activity not observed under monoculture conditions. Secondary evaluation confirmed that representative pairs, including JH077-SW175 and HH037-KS144, produced clear zones of inhibition against *S. aureus* only under co-culture conditions. Each isolate cultivated alone showed little or no inhibitory activity. Crude extracts, fractions, and subfractions derived from these pairs retained antibacterial activity in the spot-on-lawn assay. LC-MS/MS-based dereplication indicated that the active features did not match known antibiotics, suggesting previously undescribed chemical entities. Taken together, these results demonstrate that TAPA is an accessible and reusable platform for the high-throughput detection of co-culture-induced antibacterial activity and provides a practical entry point for discovering novel antibacterial natural products.

## Introduction

Microbial co-culture has emerged as one of the most promising strategies to discover novel bioactive natural products [1–3]. Cultivating two or more microorganisms together can artificially recreate the ecological competition and chemical crosstalk that naturally occur among microorganisms in their native habitats. This process can activate silent or cryptic biosynthetic gene clusters that remain transcriptionally inactive under standard monoculture conditions, or induce chimeric biosynthesis [4–6]. Several distinct mechanisms can drive this co-culture-induced microbial interaction: diffusible small-molecule signals or precursors, direct cell-to-cell contact, and competitive chemical-defense responses such as antibiotic or quorum-sensing molecule production [4, 6–8]. These interactions have led to the discovery of numerous co-culture-specific secondary metabolites, including antibiotics not detected in monoculture conditions. A well-documented case involves the marine invertebrate-associated bacterium *Micromonospora* sp. WMMB235. When co-cultured with the mycolic acid-containing bacterium *Rhodococcus* sp. WMMA185, it produced the glycosylated anthracycline antibiotic keyicin, a compound undetectable in either monoculture. Keyicin production persisted even when the two strains were physically separated by a dialysis membrane, which pointed to a diffusible chemical signal as the trigger for its induction. The compound also showed selective antibacterial activity against Gram-positive bacteria [9].

Conventional co-culture-based discovery, however, has practical and technical limitations that hinder its adoption in high-throughput antibiotic screening pipelines [5, 10]. Differences in growth rate between paired strains frequently result in one organism overgrowing and outcompeting the other before meaningful chemical interaction can occur [10, 11]. Optimizing co-culture conditions adds a further layer of difficulty: medium composition, inoculation ratio, temperature, and incubation period. As these parameters alter population balance, interaction outcomes, and metabolite production in a strain-pair-dependent manner, screening a large library becomes labor-intensive and resource-consuming [10, 12, 13].

Several innovative strategies have been developed to exert finer control over microbial interactions. Physical separation approaches such as encapsulation in alginate hydrogel beads [14, 15], microfluidic droplet-based platforms [16, 17], and membrane-separation systems (*e.g.*, Transwell inserts or dialysis chambers) [5, 18] protect slower-growing strains from overgrowth by faster-growing partners while permitting metabolite exchange; microfluidic platforms, in particular, enable high-throughput isolation of interacting pairs at the nanoliter scale. Although advanced platforms have enabled detailed analysis of microbial interactions, many require specialized instrumentation, technical expertise, and labor-intensive workflows, which limits their throughput and accessibility for routine implementation in academic laboratories [19, 20]. Co-cultivation and pathogen growth-inhibition screening are also typically run as separate, sequential procedures rather than as steps within a single assay, which further complicates integration.

To overcome these constraints, we developed the tunneled agar plate assay (TAPA), which provides spatially separated spaces for co-cultured microorganisms while permitting chemical communication through metabolite diffusion across a shared agar matrix. This 96-well plate-format assay further accommodates growth inhibition screening against pathogenic bacteria within the same plate, making it amenable to high-throughput screening and automation [21]. The plate itself is custom-engineered from non-toxic, autoclavable materials and fabricated by 3D printing, making it reusable and inexpensive to manufacture. Several of these advantages overlap with those already validated for the dual-sided agar plate assay (DAPA), which similarly demonstrated reliable antibacterial screening for single strains [20]. Building on this foundation, the present study describes the design and proof-of-concept validation of TAPA and applies it to a co-culture screen of an environmental bacterial library against pathogenic bacteria, identifying co-culture-specific antibacterial activity not observed under monoculture conditions.

## Materials and Methods

### Chemical Reagents, Strains, Media, and Culture Conditions

MTT [3-(4,5-dimethylthiazol-2-yl)-2,5-diphenyltetrazolium bromide] was purchased from Rhawn Reagent (China). *S. aureus* ATCC 6538, *E. coli* TOP10, and *P. aeruginosa* ATCC 15692 were kindly provided by Prof. Chang-Ro Lee at Myongji University. Mueller-Hinton agar (MHA) medium (Becton, Dickinson andCo., USA) was used for general cultivation of bacteria. Environmental bacteria were incubated at 30°C in 2 days mostly and pathogenic bacteria were incubated overnight at 37°C.

### TAPA Plate Design

The TAPA plate was modeled using Tinkercad software (Autodesk, USA) for subsequent 3D printing. Plates were fabricated either from BioMed Clear Resin V1 (Formlabs, USA) using a Form 4B (Formlabs) or from PAHT-CF filament (Bambu Lab, China) using P2S (Bambu Lab). The external dimensions of the plate were 124.0 × 81.0 × 15.0 mm. Two types of TAPA plates were designed with either round-shaped or fan-shaped wells.

### TAPA Growth Inhibition Assay

One layer (1,300–1,500 µL/group of MHA containing 1.5% agar) of solid nutrient medium was utilized in each group (4 wells per group) of the TAPA plate. Pouring agar into wells was performed by hand using reverse pipetting technique. After the agar was solidified, bacterial isolates for the co-cultivation were inoculated on the nutrient medium layer in the same group using a multi-channel pipette or plate replicator from their respective solutions after thawing the cryo-stocks from the environmental bacterial library. The TAPA plate was then covered by a sterile lid for 96-well microtiter plates (Kartell S.p.A., Italy) or by a 3D-printed autoclavable lid using PC filament (Bambu lab). The plates were placed in an incubator at 30°C for 2 days, depending on the optimal incubation period for the test bacteria. After the incubation of co-cultivation, 5 µL of each pathogen (*S. aureus*, *E. coli* and *P. aeruginosa*), suspended in sterile PBS at OD_590_ = 0.1 after overnight cultivation in MHB at 37°C, was inoculated onto the MHA layer. After an overnight incubation period at 37°C, growth inhibition of the pathogen was measured.

### MTT Assay

Following the TAPA growth inhibition assay, 100 µL of sterile PBS was added to each well containing the incubated pathogen. The agar surface was pipetted up and down 10–20 times to dislodge the cell mass and resuspend the pathogen cells in PBS. An aliquot of 50 µL of the resulting suspension was transferred to a standard 96-well plate and mixed with 50 µL of 5 mg/mL of MTT in PBS. After incubation for 30 min, 100 µL of DMSO was added, and the optical density at 590 nm (OD_590_) was measured using a microplate reader (Infinite F50, Tecan, Switzerland). After subtraction of the medium blank, growth inhibition was calculated as follows: growth inhibition (%) = [1 − (OD_590_ of the treated sample/OD_590_ of the untreated control)] × 100.

### Co-Culture Inhibition Assay

To confirm co-culture-induced antibacterial activity, a co-culture assay was performed by modifying the interspecies interaction assay [26] and the co-culture assay [19]. Briefly, 2 µL aliquots of environmental isolates were inoculated from cryo-stocks onto MHA, with each spot placed 10 mm apart. After an incubation period of 2–4 days, depending on the growth rate of the isolates, 2 µL of diluted *S. aureus* suspension (OD_590_ = 0.1), prepared from an overnight culture grown at 37°C in MHB, was inoculated 5 and 10 mm from the center of the co-cultivated isolates. The plates were then incubated overnight at 37°C, and growth inhibition was evaluated based on the formation of a zone of inhibition.

### Preparation of Cryo-Stocks of Pathogen Strains

*S. aureus*, *E. coli* and *P. aeruginosa* were inoculated on MHA medium overnight at 37°C. A colony from each inoculum was transferred to 5 mL of MHB in a 15 mL tube and incubated overnight in a shaking incubator (SI-600R; Lab Companion, Republic of Korea) at 37°C and 180 rpm. 0.5 mL of each bacterial broth was transferred into 1.5 mL tubes separately, and 0.5 mL of 40% (v/v) of sterile glycerol in water was added to the transferred broth. Then the mixtures were vortexed and stored in -80°C.

### Collection and Identification of Strain JH077, SW070, SW102 and SW175

Strains JH077, SW070, SW102 and SW175 were isolated from the sediment collected from an intertidal mudflat of Dangam-po and Baeksajang beach, located on the west coast of the Korean Peninsula in July 2025. The amplified 16S rRNA gene fragments of JH077, SW070, SW102 and SW175 (701-703 bp, respectively) were sequenced by Sanger sequencing (Macrogen Inc., Republic of Korea). BLASTN analysis showed that strain JH077 shared 97.2% sequence identity with *Brevibacterium epidermidis* NBRC 14811, SW070 shared 99.9% sequence identity with *Enterococcus mundtii* DSM 4838, SW102 shared 99.4% sequence identity with *Shigella dysenteriae* SWHEFF and strain SW175 shared 98.9% sequence identity with *Klebsiella variicola* F2R9T.

### Cultivation, Extraction and Fractionation of Strain JH077, SW070, SW102 and SW175

The two strains of each pair were alternately streaked in parallel on the Mueller-Hinton agar medium, and were incubated for 2 days. Then the agar containing the cultured bacteria was cut into pieces, transferred to an empty flask, and extracted with acetone overnight under shaking. After filtration through Advantec 2 filter paper (Toyo Roshi Kaisha, Ltd., Japan), the solution was evaporated in a centrifugal evaporator (CVE-3000; EYELA, Japan). The dried residue was redissolved in MeOH and subsequently fractionated by column chromatography on Sephadex LH-20 resin, yielding 10 fractions. The bioactive fractions were separated either by silica gel thin-layer chromatography (TLC) on silica gel 60 F254 plates (Merck KGaA, Germany) using ethyl acetate/*n*-hexane (5:5, v/v) as the mobile phase or by preparative reversed-phase high-performance liquid chromatography (HPLC) using an LC-Forte/R system (YMC Co., Ltd., Japan) equipped with a YMC-Pack ODS-A C18 column (10 × 250 mm, 5 mm). For preparative HPLC, separation was performed at 1.5 mL/min using a linear gradient of 50% to 100% solvent B over 30 min, followed by isocratic elution with 100% solvent B for 30 min (solvent A, water; solvent B, MeOH), yielding 45 subfractions.

## Results

### TAPA Plate Design

The tunneled agar plate (TAPA plate) was 3D-printed from non-toxic resin or filament, designed in a 96-well format, and made compatible with high-throughput automation (Fig. 1A and 1B). Each well connects via a tunnel to three others, and this group of four wells-24 groups per 96-well plate-forms the basic screening unit. Once molten agar is poured and allowed to solidify, the medium within a group forms a single continuous piece, yet the agar surface in each well remains spatially separated; metabolites can migrate between wells, but bacterial cells cannot (Fig. 1C and 1D). TAPA is primarily designed for screening the inhibitory activity of environmental microorganisms against pathogenic bacteria under co-culture conditions. Two to three of the four wells in a group, for instance, can be inoculated with different test bacteria and incubated so that their metabolites could diffuse through the agar; this diffusion enables chemical communication between neighboring cultures and can induce secondary metabolites-including antibiotics not seen in monoculture (Fig. 1C). The platform also accommodates single-strain screening: when one well in a group is inoculated with a single test bacterium, its inhibitory activity can be evaluated simultaneously against up to three different pathogenic strains (Fig. 1D). Because any well can independently host an environmental isolate, biological or chemical elicitor, or a pathogen, the plate allows a wide range of combinations to be screened for growth inhibition through metabolite diffusion alone.

### Proof of Concept of TAPA

Diffusion of bacterial metabolites, especially antibacterial compounds, through agar medium has been demonstrated using various techniques including disk diffusion, the Lutri plate, the Janus plate, a transwell system embedded in an agar plug, the agar-plug cultivation system and the DAPA [20, 22–25]. We validated this same principle in the TAPA system using three antibiotics-ampicillin, aztreonam, and imipenem -tested across a concentration range of 0.01–6.25 µg/mL against *E. coli*, *P. aeruginosa*, and *S. aureus*. The antibiotic solution was added to the agar in one well of each group, and the plate was incubated overnight at 30°C to allow diffusion, mimicking the release of bacterial specialized metabolites during incubation. Each of the three pathogens was then inoculated into one of the remaining wells and incubated overnight at 37°C; growth inhibition was assessed both visually and quantitatively, the latter by measuring OD_590_ after treatment with the redox dye MTT (3-(4,5-dimethylthiazol-2-yl)-2,5-diphenyltetrazolium bromide). Diffusion of the combined antibiotics, ampicillin and aztreonam, successfully inhibited the growth of all three pathogens, whereas no inhibition was observed in the negative control containing agar medium alone (Fig. 2A and 2B). MIC values obtained using TAPA were of a comparable order of magnitude to those determined by broth microdilution, though somewhat higher overall. This difference likely reflects the assay format itself rather than any inconsistency of the method (Supplementary Fig. S1).

We next asked whether TAPA plates could detect growth inhibition in competition assays involving environmental isolates. Strains exhibiting inhibitory activity against at least one of the three pathogens were first screened from our in-house bacterial library using the cross-streak method, and a subset of the bioactive isolates was subsequently tested in the TAPA system against the same three pathogens. Metabolites diffusing from isolate KS094 inhibited the growth of all three pathogens, whereas isolate KS138 exhibited selective inhibition against *E. coli* and *S. aureus* (Fig. 2C and 2D). Quantitative measurement of growth inhibition via the MTT assay matched these visual observations closely (Fig. 2E-2I), which supports the reliability of TAPA for detecting diffusion-mediated growth inhibition across multiple pathogens at once.

### TAPA Screening of an Environmental Bacterial Library

From an in-house library of environmental bacterial isolates, we selected 83 isolates for TAPA-based co-culture screening. Each isolate displayed a distinct colony morphology but showed no inhibitory activity against *S. aureus* in the preliminary cross-streak assay. Testing every possible pairwise combination of these 83 isolates-including self-combinations and reciprocal duplicates-generated 6,889 individual assays in total. For the co-culture assays, the environmental isolates were first inoculated from cryo-stocks onto MHA in the TAPA device and incubated for 2 days, after which *S. aureus* was introduced into the adjacent well; growth inhibition was assessed visually following an additional overnight incubation (Fig. 3A-3G). Among the 6,889 combinations, 106 co-culture pairs-the screening hits-exhibited inhibitory activity against *S. aureus*. This screening effort spanned two weeks, with the 83 TAPA plates reused across four separate rounds of assays, and the full results are summarized in Supplementary Table S1.

### Secondary Evaluation of TAPA-Identified Co-Culture Hits

We turned next to confirming that the antibacterial activity identified by TAPA screening genuinely depended on co-culture, rather than reflecting an artifact of the screening format itself. The bioactive co-culture combinations were re-evaluated using a co-culture inhibition assay, while each isolate was cultivated separately as a control (Fig. 4A and 4B). Co-cultivation of isolates JH077 and SW175, for example, produced inhibitory activity against *S. aureus* (Fig. 4E), yet inhibition disappeared entirely when either isolate was cultivated alone (Fig. 4C and 4D). Co-cultivation of HH037 and KS144 similarly produced a clear zone of inhibition against *S. aureus* (Fig. 4H). HH037 alone did not form a distinct zone of inhibition but did reduce the cell density of *S. aureus*, pointing to weak intrinsic activity (Fig. 4F); KS144 alone showed none at all (Fig. 4G). 17 of the 106 screening hits (16.0%) could not be evaluated using the conventional co-culture inhibition assay because fast-growing, swimming, or swarming isolates overgrew their partners and the adjacent pathogen spots before a zone of inhibition could be assessed (Fig. S2A and S2B).

To determine whether antibiotic metabolites were responsible for the observed inhibition, we randomly selected a subset of the active pairs and co-inoculated them on MHA in an alternating pattern, followed by incubation for 2 days (Fig. S3A). The resulting crude extracts were tested against *S. aureus* in the spot-on-lawn assay, and all six pairs tested showed activity (Fig. S3B- S3D). Two of these-JH077-SW175 and SW070-SW102 pairs-were carried forward for fractionation by column chromatography on Sephadex LH-20 resin. The collected fractions were evaporated, reconstituted in methanol (5 mg/mL), and evaluated for activity; a single fraction from each set produced a zone of inhibition against *S. aureus* (Fig. S3E and S3F). These bioactive fractions were fractionated further by reverse-phase HPLC or silica gel TLC, and a subfraction from each sample retained inhibitory activity when tested in the same manner (Fig. S3G and S3H).

## Discussion

The TAPA system was developed to address several limitations of conventional co-culture assays and to enable more efficient screening for pathogen-inhibitory activity. By spatially separating the growth areas of two or more microorganisms, the system avoids the rapid overgrowth by dominant strains that so often derails co-culture experiments. Its agar-based format also permits diffusion of microbial metabolites, which facilitates chemical communication between strains and enhances co-culture-associated effects. Although TAPA is designed primarily for co-culture screening, the platform works equally well for antibacterial screening of single strains. A further practical advantage is that pathogen growth inhibition can be assessed visually, without fluorescence-labeled transformants or other reporter strains. When visual inspection is difficult or a quantitative readout is needed, the MTT-based assay remains available as an alternative. In this study, the assay was performed using only one type of nutrient agar medium, but an agar-overlay format could be adapted to run multiple agar media in parallel, as previously shown for DAPA, or to substitute media better suited for the target pathogen.

The secondary evaluation of the screening hits provided direct evidence that the observed antibacterial activity was co-culture-specific rather than an intrinsic property of either isolate alone. In the co-culture inhibition assay, paired isolates such as JH077-SW175 and HH037-KS144 produced a clear zone of inhibition against *S. aureus*, while each isolate cultivated individually showed little to no activity, confirming that sufficient antibacterial activity emerged only under co-culture conditions. This conclusion was also supported by chemical analysis: crude extracts and their fractions and subfractions from the co-cultivated pairs retained inhibitory activity against *S. aureus* in the spot-on-lawn assay, providing evidence that the inhibition was mediated by specialized metabolites induced through co-culture interaction as the underlying cause. When the active subfractions were subjected to LC-MS/MS-based dereplication against the GNPS, SIRIUS, and NP Atlas databases, none of the detected features matched known antibiotics (data not shown), suggesting that the co-culture-induced antibacterial compounds may represent novel chemical entities. Taken together, these results indicate that TAPA is not only effective for detecting co-culture-dependent growth inhibition but also serves as a practical entry point for discovering antibacterial natural products that have not yet been described.

The co-culture inhibition assay used for secondary evaluation, however, also exposed a limitation of conventional, non-spatially separated formats. Several strain pairs simply could not be evaluated, as one partner grew across the entire plate before a zone of inhibition could be evaluated. This observation highlights, by direct comparison, the advantage of the spatial separation implemented in the TAPA design. TAPA confines each isolate to a physically distinct well while still permitting metabolite diffusion through the connected agar matrix. This spatial separation avoids the overgrowth-related failures that undermined the conventional assay, an advantage that becomes especially valuable when screening strain pairs with markedly different growth rates. During secondary validation, this problem may be mitigated by reducing the inoculum size or preincubation time of rapidly growing isolates, staggering inoculation according to strain-specific growth rates, or testing cell-free extracts when direct plate-based assessment is not feasible.

A limitation of the TAPA platform is that it may be poorly suited to combinations whose co-culture effects depend on direct physical contact rather than diffusible signals. In addition, when strains with markedly different growth rates are combined, determining an appropriate incubation period becomes an important consideration in screening large strain libraries. Contamination was rarely observed with resin-printed plates, but occasional contaminant growth did occur in filament-printed plates during the screening. This is plausibly caused by microscopic voids generated during filament-based 3D printing. Such voids may allow contaminating spores to enter during washing, and the heat-resistant filament’s internal structure may limit how effectively sterilization reaches them. Soaking the filament-printed plates in 5% bleach after washing, then drying them thoroughly before use, substantially reduced contamination.

Because TAPA is an agar-based platform, it should be applicable not only to bacteria but also to other microorganisms, including fungi. Its utility need not stop at antibiotic discovery, either-studies of microbial interactions in environmental ecosystems, analyses of gut microbiome interactions, and screening for anti-protozoal or antiviral activity all seem like reasonable extensions of the same basic design.

## Supplemental Materials

Supplementary data for this paper are available on-line only at http://jmb.or.kr.



## Figures and Tables

**Fig. 1:**
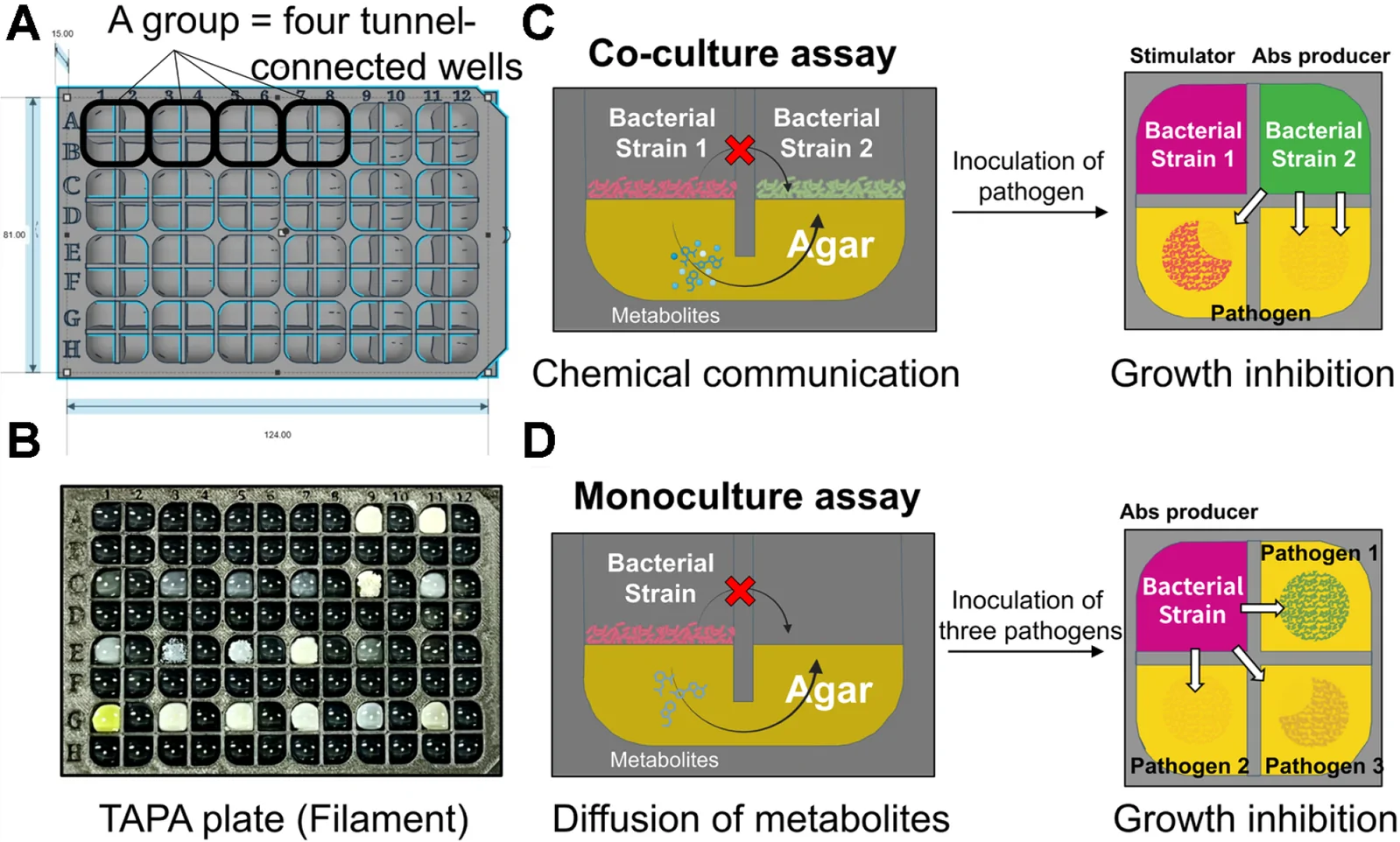
Schematic design and working principle of the TAPA plate. The TAPA plate is a 96-well format comprising 24 groups of four tunnel-connected wells (**A**), fabricated via 3D printing using non-toxic, autoclavable materials, with each environmental isolate inoculated into a single well per group (**B**). The tunnel-connected wells allow free diffusion of metabolites across the shared agar matrix, enabling both inter-isolate chemical communication and growth-inhibition screening against a target pathogen within the same plate (**C**), and permit simultaneous testing against up to three different pathogens in the monoculture assay.

**Fig. 2:**
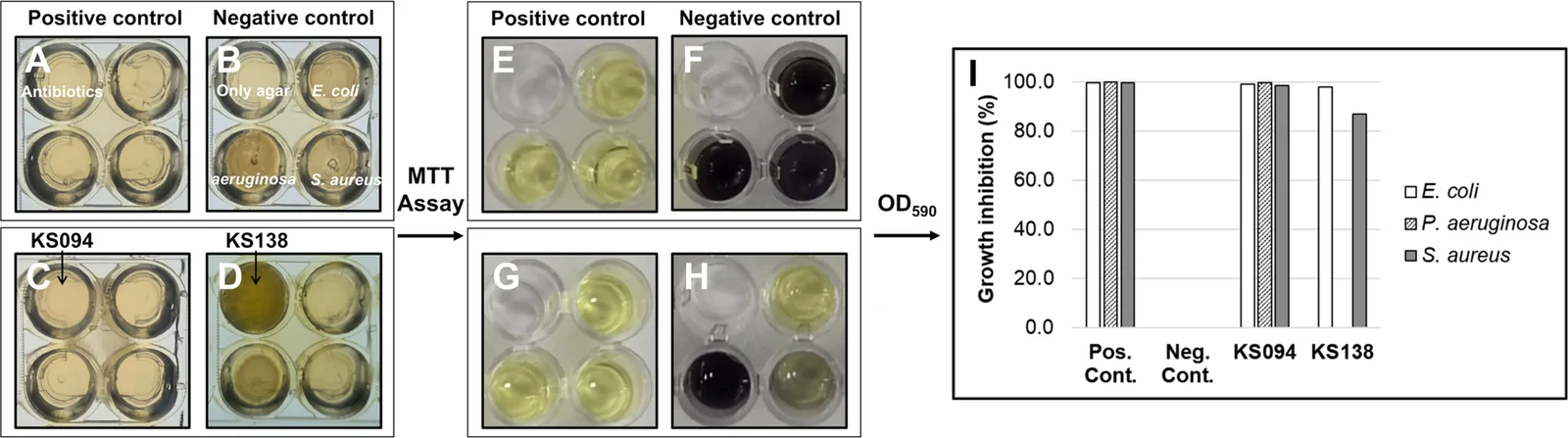
Proof of concept of the TAPA. In each group, one well (left top) received a test sample, either combined antibiotics, agar medium alone, or an environmental isolate, while the remaining three wells were individually inoculated with *E. coli*, *P. aeruginosa* and *S. aureus*. Diffusion of combined antibiotics ampicillin and aztreonam inhibited the growth of all three pathogens (**A**), whereas the negative control shows no inhibition (**B**). Metabolites diffusing from isolate KS094 inhibited the growth of all three pathogens (**C**), whereas isolate KS138 exhibited selective inhibition (**D**). The corresponding MTT assay results are shown in (**E-H**), and the OD_590_-based measurements provided a quantitative assessment ot the pathogen growth-inhibition levels (**I**).

**Fig. 3:**
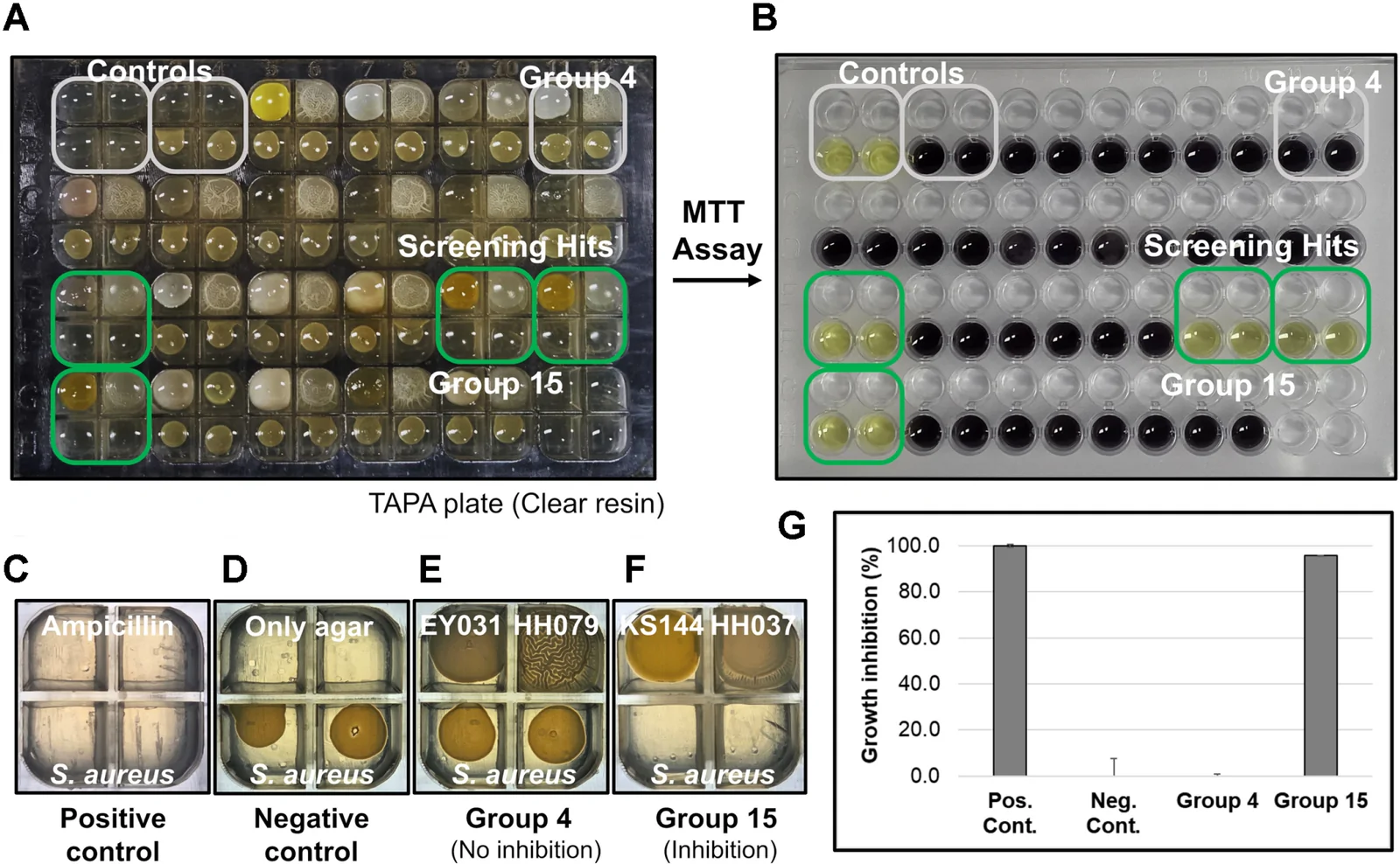
Co-culture TAPA screening against *S. aureus*. A representative TAPA screening result (**A**) and the corresponding MTT assay result (**B**) show growth inhibition against *S. aureus*. Ampicillin (**C**) and nutrient agar alone (**D**) served as the positive and negative controls, respectively. Visible growth indicates no inhibition (**E**), whereas its absence indicates inhibition (**F**). The MTT assay, used as an orthogonal detection method, yielded results consistent with visual observation (**G**).

**Fig. 4:**
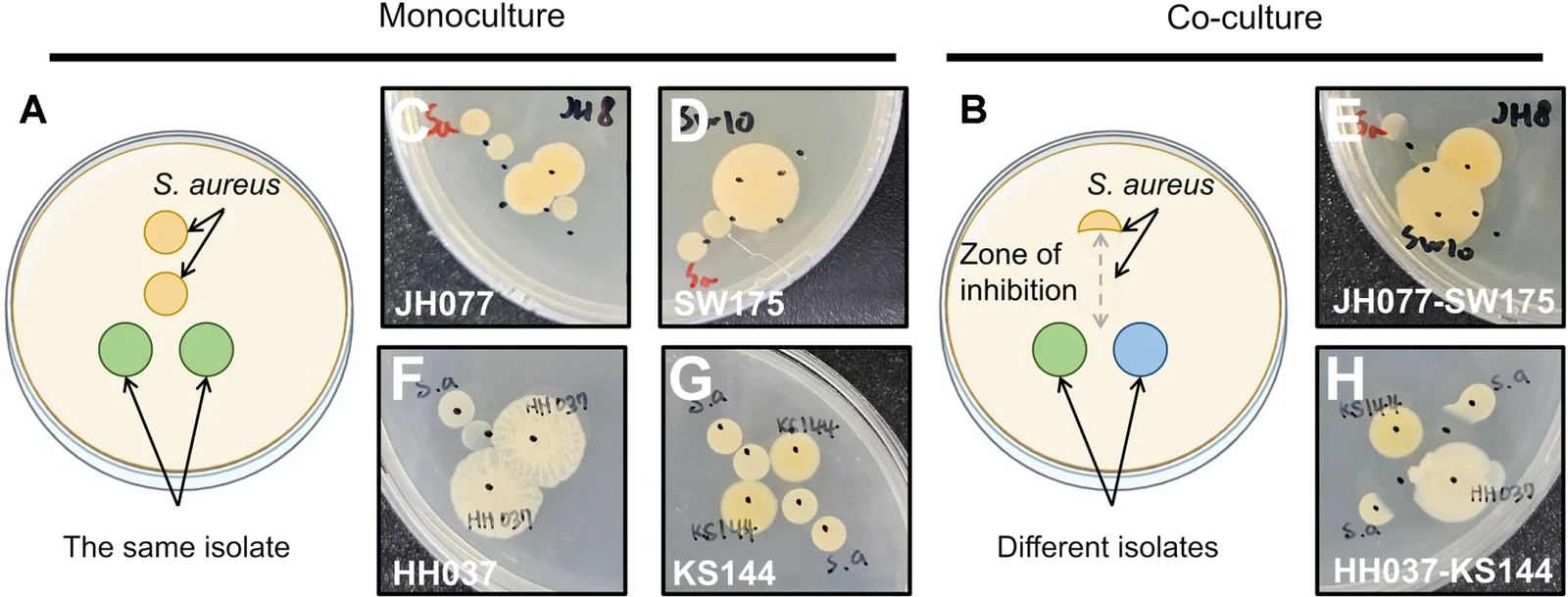
Confirmation of co-culture-induced antibacterial activity against *S. aureus*. Schematic representation of the monoculture (**A**) and co-culture (**B**) inhibition assay used to evaluate environmental isolates against *S. aureus*. Neither JH077 (**C**) nor SW175 (**D**) alone inhibited *S. aureus*, whereas their co-culture produced a clear zone of inhibition (**E**). HH037 (**F**) alone showed weak inhibition and KS144 (**G**) showed none in monoculture, yet their co-culture produced a distinct zone of inhibition (**H**).
