# Supplementary material for: Development of the Tunneled Agar Plate Assay (TAPA) for Microbial Co-culture Screening of Antibacterial Activity against Pathogenic Bacteria
Source: J Microbiol Biotechnol. 2026 Oct 14;36:e2608043. doi: 10.4014/jmb.2608.08043 (PMC13720290; doi:10.4014/jmb.2608.08043)
Supplement: Supplementary file 1 [file jmb-36-e2608043-supple.pdf]

J. Microbiol. Biotechnol. 2026. 36: e2608043

https://doi.org/10.4014/jmb.2608.08043

Supplementary Figures and Table

Development of the Tunneled Agar Plate Assay (TAPA) for Microbial Co-culture Screening of Antibacterial Activity against Pathogenic Bacteria

Gyeongsik Shin†, Hyuntae Kim†, Yujeong Kim†, Junho Lee, Juyoung Kang, Eunyeong Yun, Taeseop Song, Seunghyeon Ha, Jinhyeok Hur, Haein Lee, Sol Kang, Chaeyeon Kim, Siwon Choi, Jeongmin Park, Garam Kim, and Jeongho Lee*

Department of Biosciences and Bioinformatics, Myongji University, Yongin 17058, Republic of Korea

*Corresponding Author: JL, E-mail: jeongholee@mju.ac.kr

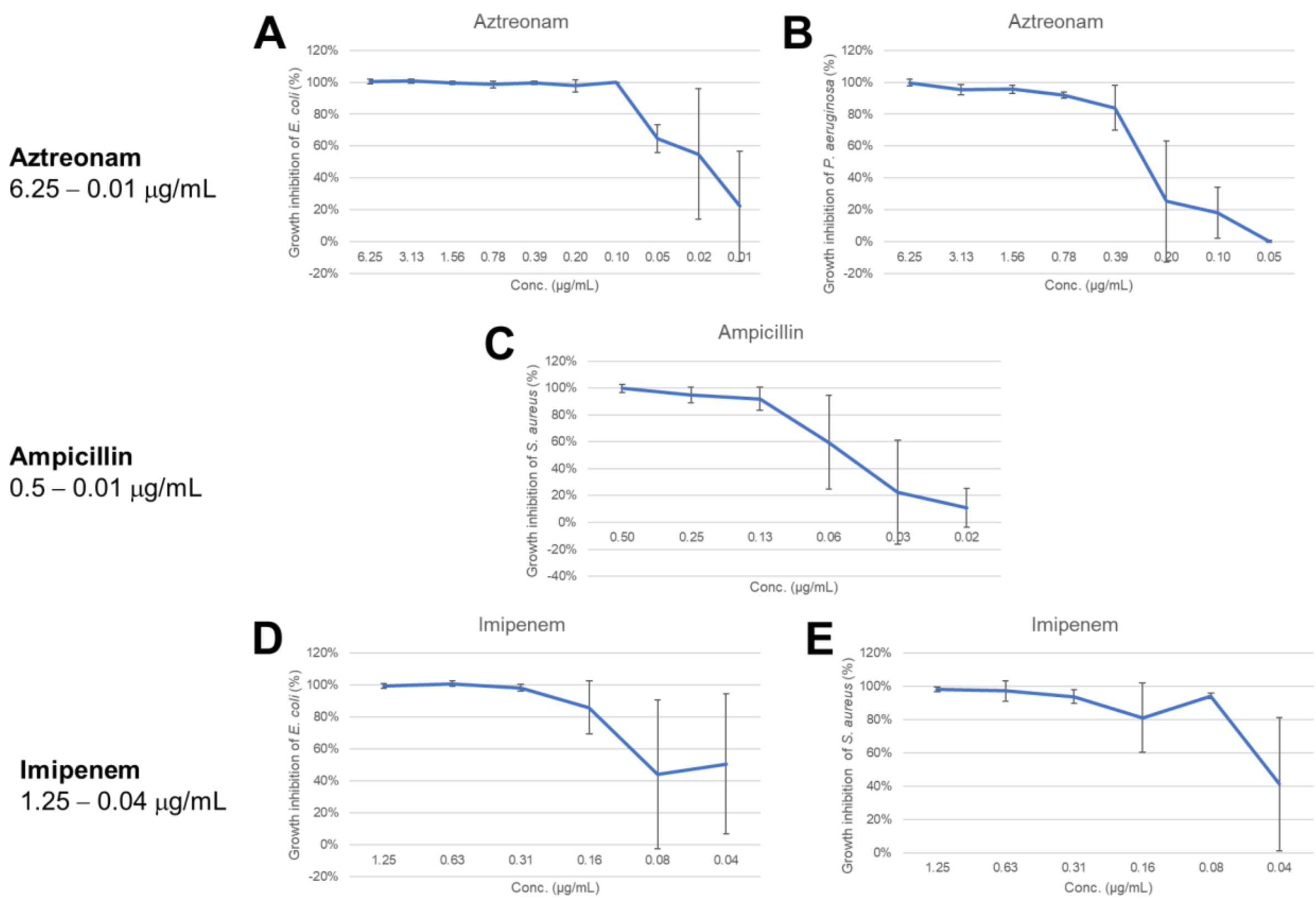

Fig. S1. Validation of TAPA using known antibiotics.

Serial dilutions of antibiotics were tested for inhibitory activity against the indicated pathogens using the TAPA system (n = 3). In the observed inhibition, Aztreonam exhibited the lowest inhibitory concentrations of 0.10 against *E. coli* (**A**) and 0.78 μg/mL against *P. aeruginosa* (**B**), which were of a comparable order of magnitude to the reported MIC values of 0.06 for *E. coli* [1] and 4 μg/mL for *P. aeruginosa* PAO1 [2], as determined by the broth-based serial dilution assay. Ampicillin exhibited the lowest inhibitory concentrations of 0.13 μg/mL against *S. aureus* (**C**), consistent with the reported MIC of ≤0.25 μg/mL [3]. Imipenem exhibited the lowest inhibitory concentrations of 0.31 against *E. coli* (**D**) and 0.08 μg/mL against *S. aureus* (**E**), respectively, which were within a broadly similar range to the reported MIC was 0.25 [4] and 0.015–0.06 μg/mL [5] (*S. aureus* ATCC 29213). Data are presented as the mean ± SD of three independent measurements.

Table S1. The results of the TAPA screening of all 6,889 combinations of environmental isolates against *S. aureus*

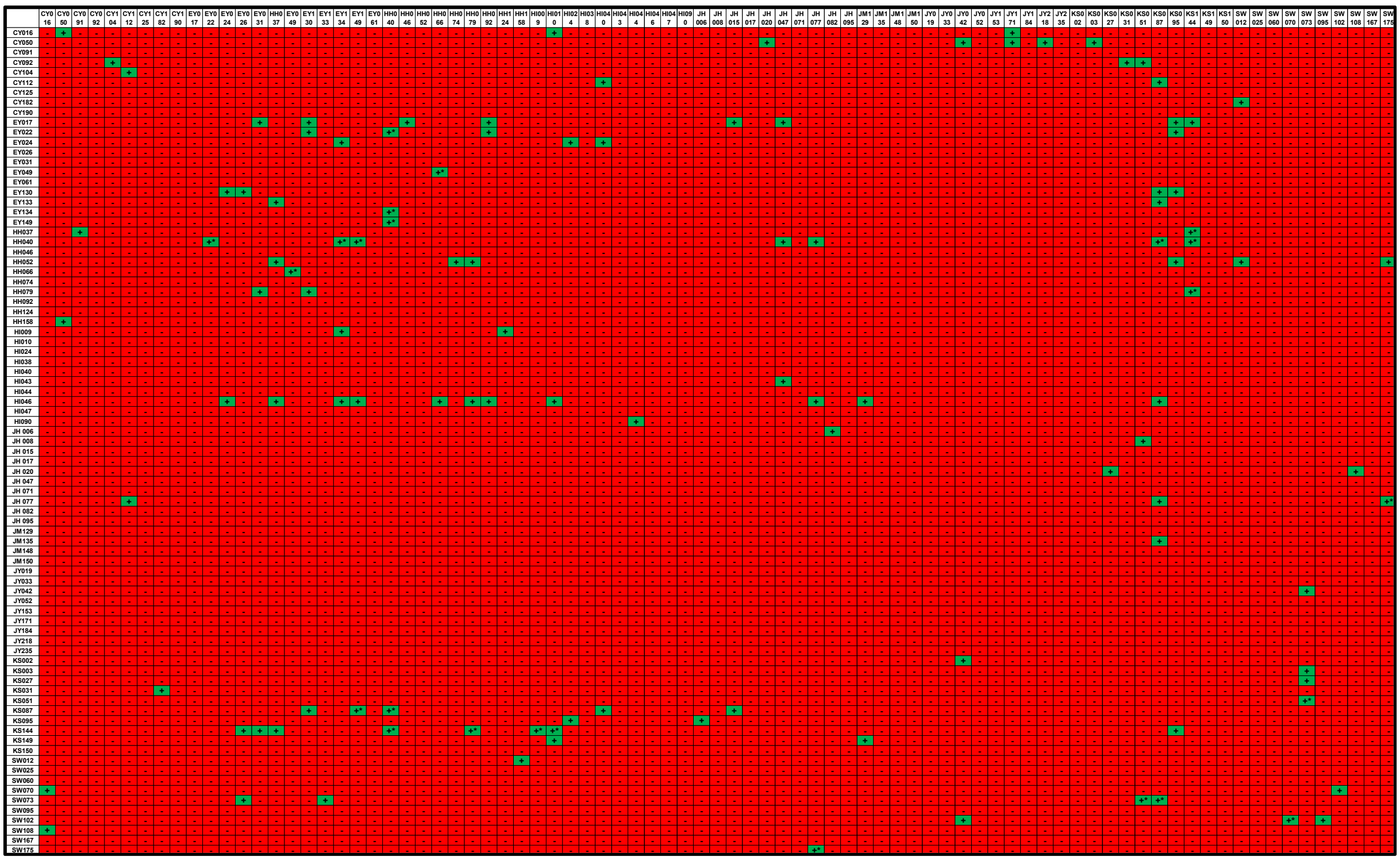

All pairwise combinations of the 83 isolates, comprising 6,889 individual co-culture assays, are shown. Green cells marked "+" indicate growth inhibition against *S. aureus*, whereas red cells marked "-" indicate no inhibition. "+*" in green colored cell represents growth of inhibition against *S. aureus* in the re-test because of the contamination in the first screening.

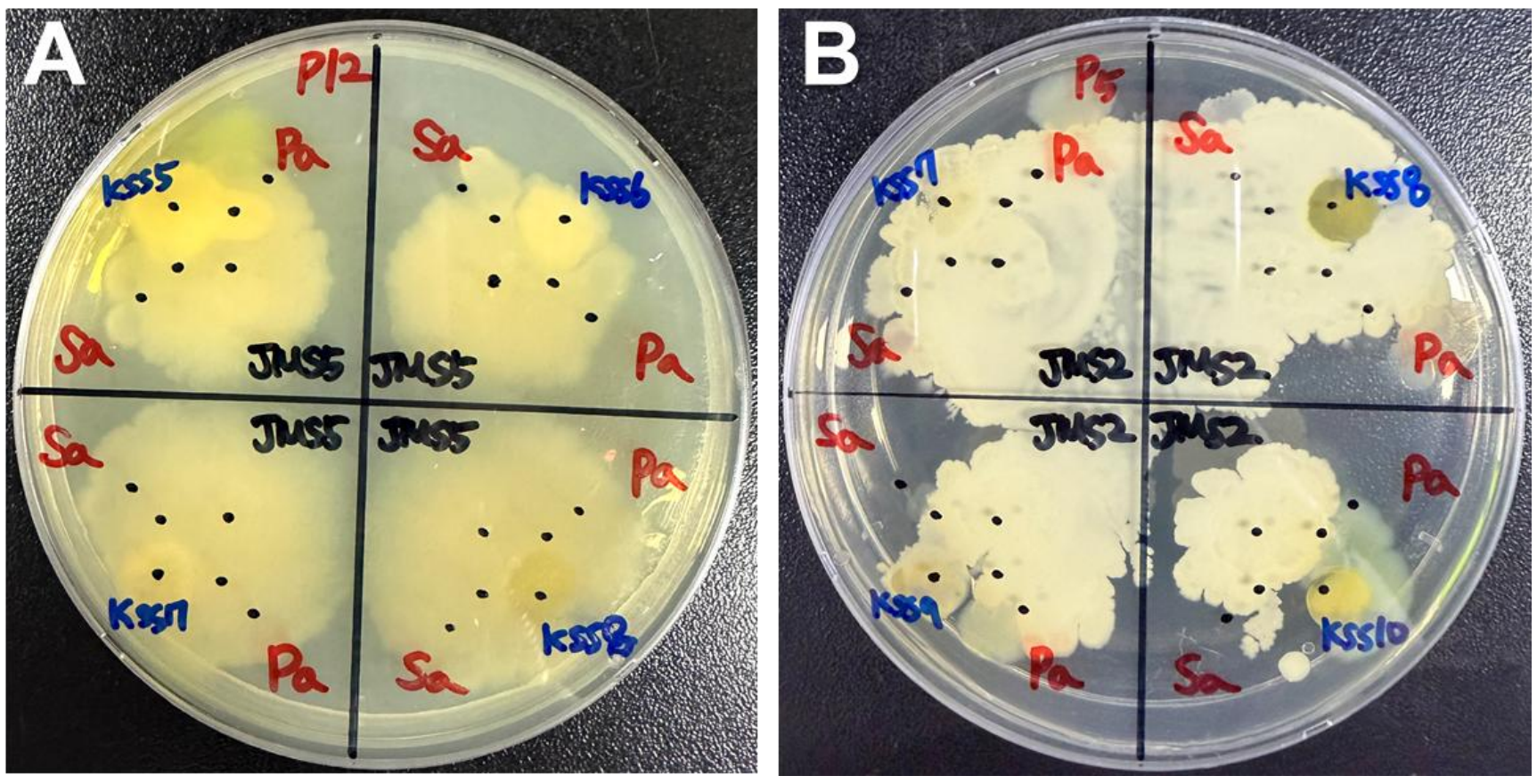

Fig. S2. Representative failure of the co-culture inhibition assay due to overgrowth by a fast-growing isolate.

Co-culture inhibition assays were performed using JMS5 (**A**) and JMS2 (**B**) as the central test strain, each paired with multiple partner isolates and the pathogens *S. aureus* (Sa) and *P. aeruginosa* (Pa) spotted around the center. In both plates, the overgrowth of JMS5 and JMS2 rapidly overtook the surrounding spots, obscuring the individual colonies of the co-cultivated isolates and pathogens before a clear zone of inhibition could be assessed, illustrating the overgrowth-related limitation of the conventional co-culture inhibition assay. Among the 106 co-culture pairs, 17 pairs (16%) showed this overgrowth issue during the secondary evaluation.

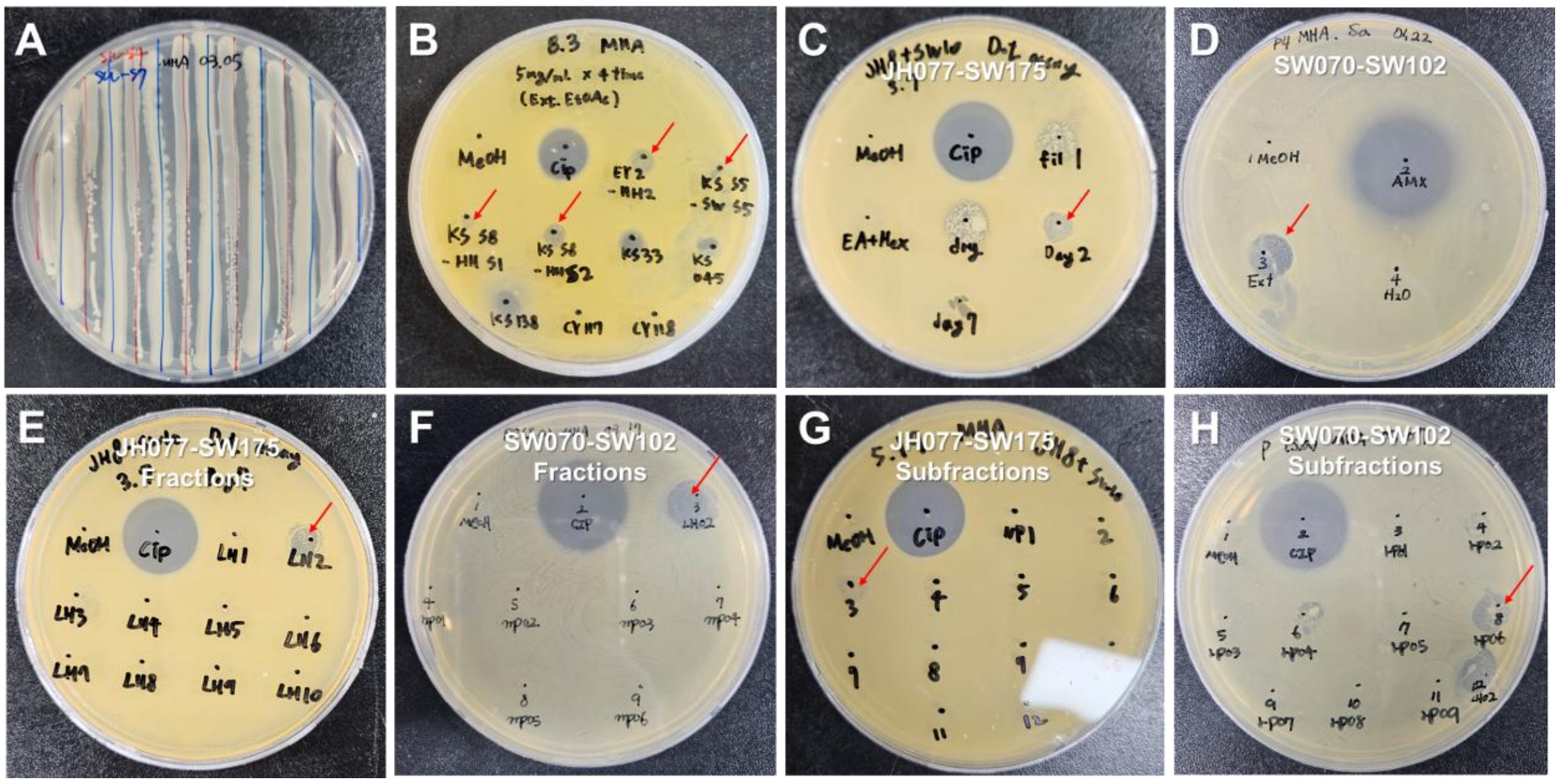

Fig. S3. Bioassay-guided fractionation of co-culture extracts active against *S. aureus*.

Active co-culture combinations were co-inoculated on MHA in an alternating streak pattern and incubated for 2 days to generate crude extracts (**A**). The resulting crude extracts from multiple co-culture pairs were screened for antibacterial activity against *S. aureus* using the spot-on-lawn assay, with ciprofloxacin as the positive control; extracts from the pairs EY022-HH040, KS051-SW073, HH037-KS144, HH040-KS144 (**B**), JH077-SW175 (**C**) and SW070-SW102 (**D**) exhibited clear zones of inhibition (arrows). The crude extracts were then fractionated by column chromatography on Sephadex LH-20 resin, and a single active fraction was identified from each of the JH077-SW175 (**E**) and SW070-SW102 (**F**) extracts (arrows). These bioactive fractions were further separated by reverse-phase HPLC or silica gel TLC, yielding a single active subfraction from each of the JH077-SW175 (**G**) and SW070-SW102 (**H**) samples (arrows), which retained inhibitory activity against *S. aureus* in the spot-on-lawn assay.

References

1. Sadek M, Juhas M, Poirel L, Nordmann P. 2020. Genetic Features Leading to Reduced Susceptibility to Aztreonam-Avibactam among Metallo-β-Lactamase-Producing Escherichia coli Isolates. *Antimicrob. Agents Chemother.* **64**: e01659-20.
2. Ropy A, Cabot G, Sánchez-Diener I, Aguilera C, Moya B, Ayala JA, *et al.* 2015. Role of Pseudomonas aeruginosa Low-Molecular-Mass Penicillin-Binding Proteins in AmpC Expression, β-Lactam Resistance, and Peptidoglycan Structure. *Antimicrob. Agents Chemother.* **59**: 3925–3934.
3. Mazzantini D, Massimino M, Calvigioni M, Rossi V, Celandroni F, Lupetti A, *et al.* 2024. Anti-staphylococcal activity of a polyphenol-rich citrus extract: synergy with β-lactams and low proficiency to induce resistance. *Front. Microbiol.* **15**: 1415400.
4. Jousset AB, Rosinski-Chupin I, Takissian J, Glaser P, Bonnin RA, Naas T. 2018. Transcriptional Landscape of a blaKPC-2 Plasmid and Response to Imipenem Exposure in Escherichia coli TOP10. *Front. Microbiol.* **9**: 2929.
5. Brown-Elliott BA, Killingley J, Vasireddy S, Bridge L, Wallace RJ. 2016. *In Vitro* Comparison of Ertapenem, Meropenem, and Imipenem against Isolates of Rapidly Growing Mycobacteria and Nocardia by Use of Broth Microdilution and Etest. *J. Clin. Microbiol.* **54**: 1586–1592.
